# Supplementary material for: Non-Saccharomyces Yeasts from Organic Vineyards as Spontaneous Fermentation Agents
Source: Foods. 2023 Oct 2;12(19):3644. doi: 10.3390/foods12193644 (PMC10572797; doi:10.3390/foods12193644)
Supplement: Supplementary file 1 [file foods-12-03644-s001.zip › Figure S1.pdf]

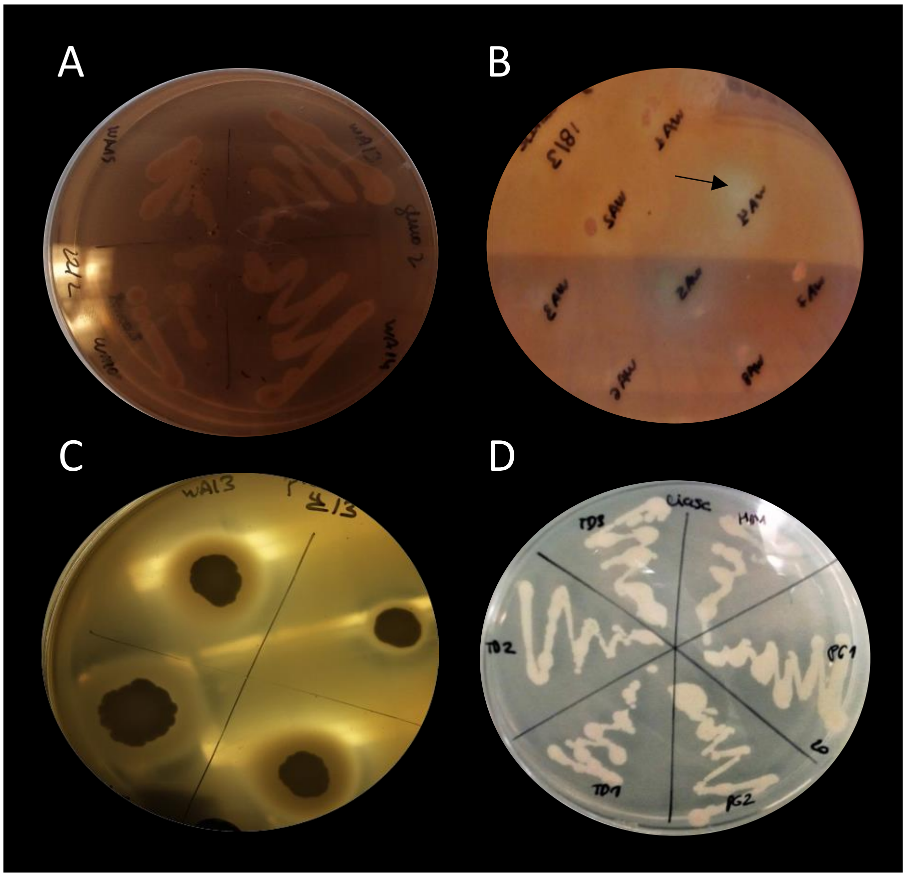

Figure S1. Enzymatic activities plates. Dark-black cultures were considered positive β-glucosidase activity (A), positive β-glucanase activity showed a clear halo on the surface after removing the colonies (B), a clear halo around the colonies reported a positive protease activity (C) and β-lyase activity was considered positive when the growth of the isolates was significant (D).
