# Supplementary material for: Non-Saccharomyces Yeasts from Organic Vineyards as Spontaneous Fermentation Agents
Source: Foods. 2023 Oct 2;12(19):3644. doi: 10.3390/foods12193644 (PMC10572797; doi:10.3390/foods12193644)
Supplement: Supplementary file 1 [file foods-12-03644-s001.zip › Table S1.pdf]

Table S1. Number of non-*Saccharomyces* isolates analysed in this work, depending on the vineyard, vintage and fermentation stage.

**Fermentation stages**	**Vineyard 1**		**Vineyard 2**		**Vineyard 3**		Total
	**1st vintage**	**2nd vintage**	**1st vintage**	**2nd vintage**	**1st vintage**	**2nd vintage**	
CM	4	0	4	8	12	9	37
RM	0	0	0	2	4	6	12
SF	1	0	0	0	0	0	1
TF	0	0	0	0	1	0	1
EF	1	0	0	2	1	0	4
Total	6	0	4	12	18	15	**55**

Fermentation stages: freshly crushed grape must, CM; racked must, RM; start of fermentation, SF; tumultuous fermentation, TF; end of fermentation, EF.
