# Supplementary material for: Non-Saccharomyces Yeasts from Organic Vineyards as Spontaneous Fermentation Agents
Source: Foods. 2023 Oct 2;12(19):3644. doi: 10.3390/foods12193644 (PMC10572797; doi:10.3390/foods12193644)
Supplement: Supplementary file 1 [file foods-12-03644-s001.zip › Table S2.pdf]

Table S2. Measurement of β-lyase activity.

Torulaspora delbruckii		*Pichia guilliermondii*	
**Td01**	9.84 ± 0.03 ^{a}	**Pg01**	9.61 ± 0.02 ^{a}
**Td02**	9.86 ± 0.06 ^{a}	**Pg02**	9.55 ± 0.03 ^{b}
**Td03**	9.89 ± 0.01 ^{a}		
Hanseniaspora osmophila		***Hanseniaspora meyeri***	
**Ho01**	9.79 ± 0.09	**Hm01**	9.80 ± 0.04
Pichia kudriavzevii		***Wickerhamomyces anomalus***	
**Pk01**	9.39 ± 0.07 ^{a}	**Wa01**	9.20 ± 0.02 ^{a}
**Pk02**	9.57 ± 0.01 ^{bc}	**Wa02**	9.33 ± 0.04 ^{ab}
**Pk03**	9.46 ± 0.03 ^{ab}	**Wa03**	9.34 ± 0.06 ^{bc}
**Pk04**	9.71 ± 0.03 ^{cd}	**Wa04**	9.46 ± 0.03 ^{cde}
**Pk05**	9.72 ± 0.03 ^{d}	**Wa05**	9.46 ± 0.02 ^{de}
**Pk06**	9.67 ± 0.11 ^{cd}	**Wa06**	9.55 ± 0.01 ^{ef}
**Pk07**	9.73 ± 0.06 ^{d}	**Wa07**	9.49 ± 0.07 ^{de}
**Pk08**	9.64 ± 0.05 ^{cd}	**Wa08**	9.39 ± 0.03 ^{bcd}
**Pk09**	9.71 ± 0.00 ^{cd}	**Wa09**	9.50 ± 0.04 ^{de}
**Pk10**	9.61 ± 0.02 ^{bcd}	**Wa10**	9.57 ± 0.00 ^{ef}
**Pk11**	9.67 ± 0.05 ^{cd}	**Wa11**	9.54 ± 0.01 ^{ef}
**Pk12**	9.66 ± 0.00 ^{cd}	**Wa12**	9.76 ± 0.01 ^{g}
**Pk13**	9.61 ± 0.01 ^{cd}	**Wa13**	9.53 ± 0.04 ^{ef}
		**Wa14**	9.64 ± 0.07 ^{fg}
		**Wa15**	9.51 ± 0.07 ^{def}
		**Wa16**	9.48 ± 0.04 ^{de}

Quantification of β-lyase activity expressed as yeast growth after 48h (Log_{10}CFU/mL). Different letters in the same yeast species indicate significant differences among β-lyase activity of the isolates analysed ($p < 0.05$).
